# The Role of Human Immunodeficiency Virus–Associated Vasculopathy in the Etiology of Stroke

**DOI:** 10.1093/infdis/jix340

**Published:** 2017-07-22

**Authors:** Laura A Benjamin, Theresa J Allain, Henry Mzinganjira, Myles D Connor, Colin Smith, Sebastian Lucas, Elizabeth Joekes, Sam Kampondeni, Karen Chetcuti, Ian Turnbull, Mark Hopkins, Steve Kamiza, Elizabeth L Corbett, Robert S Heyderman, Tom Solomon

**Affiliations:** 1 Malawi-Liverpool-Wellcome Trust Clinical Research Programme, University of Malawi College of Medicine, Blantyre;; 2 Brain Infections Group, Institute of Infection and Global Health, University of Liverpool, United Kingdom;; 3 Department of Medicine, College of Medicine, University of Malawi, Blantyre;; 4 Walton Centre NHS Foundation Trust, Liverpool,; 5 NHS Borders, Melrose, and; 6 Division of Clinical Neurosciences, University of Edinburgh, United Kingdom;; 7 School of Public Health, University of the Witwatersrand, Johannesburg, South Africa;; 8 Centre for Clinical Brain Sciences, University of Edinburgh,; 9 Department of Histopathology, North Wing, St Thomas’ Hospital, London,; 10 Department of Radiology, Royal Liverpool University Hospital,; 11 Department of Radiology, Alder Hey Children’s NHS Foundation Trust, Liverpool,; 12 North Manchester General Hospital, and; 13 Royal Liverpool University Hospital, United Kingdom;; 14 Department of Pathology, College of Medicine, University of Malawi, Blantyre;; 15 Department of Clinical Research, London School of Hygiene and Tropical Medicine,; 16 Division of Infection and Immunity, University College London,; 17 Walton Centre NHS Foundation Trust, Liverpool, and; 18 Health Protection Research Unit in Emerging and Zoonotic Infections, National Institute for Health Research,Liverpool,United Kingdom

**Keywords:** stroke, vasculopathy, HIV, Africa, immune reconstitution syndrome

## Abstract

**Background:**

Human immunodeficiency virus (HIV) infection is a recognized risk factor for stroke among young populations, but the exact mechanisms are poorly understood. We studied the clinical, radiologic, and histologic features of HIV-related ischemic stroke to gain insight into the disease mechanisms.

**Methods:**

We conducted a prospective, in-depth analysis of adult ischemic stroke patients presenting to Queen Elizabeth Central Hospital, Blantyre, Malawi, in 2011.

**Results:**

We recruited 64 HIV-infected and 107 HIV-uninfected patients. Those with HIV were significantly younger (*P* < .001) and less likely to have established vascular risk factors. Patients with HIV were more likely to have large artery disease (21% vs 10%; *P* < .001). The commonest etiology was HIV-associated vasculopathy (24 [38%]), followed by opportunistic infections (16 [25%]). Sixteen of 64 (25%) had a stroke soon after starting antiretroviral therapy (ART), suggesting an immune reconstitution–like syndrome. In this group, CD4^+^ T-lymphocyte count was low, despite a significantly lower HIV viral load in those recently started on treatment (*P* < .001).

**Conclusions:**

HIV-associated vasculopathy and opportunistic infections are common causes of HIV-related ischemic stroke. Furthermore, subtypes of HIV-associated vasculopathy may manifest as a result of an immune reconstitution–like syndrome after starting ART. A better understanding of this mechanism may point toward new treatments.

## Summary

The etiologic spectrum of stroke is different in those with HIV infection compared to those without. HIV-associated vasculopathy was the commonest mechanism found. We describe an immune reconstitution syndrome–like vasculopathy among those starting antiretroviral therapy.

(See the editorial commentary by Smith, on pages 509–10.)

Stroke incidence in low- to middle-income countries is increasing, especially in young populations [1]. In many of these regions, human immunodeficiency virus (HIV) is prevalent, and younger populations are more likely to have infectious causes of stroke [2].

We recently showed that HIV infection makes a major contribution to the overall stroke burden (population attributable fraction [PAF] = 15%) in Malawi [2]. It was the second leading risk factor overall (behind hypertension), and the most important among young stroke patients (PAF = 42%). Starting antiretroviral therapy (ART) appeared to contribute to stroke risk in the very immunosuppressed, but the mechanism of this is unknown [2]. Previous reports have shown that opportunistic infections, coagulopathy, and cardiothromboembolism are important etiologies to consider [3]. In addition, HIV infection may directly lead to HIV-associated vasculopathy via inflammatory intermediaries [4]. The term “vasculopathy” is defined as intimal hyperplasia more than expected for age, and thus encompasses several pathologic phenotypes of stroke found in HIV infection, including (1) HIV-associated accelerated atherosclerosis; (2) nonatherosclerotic vasculopathy (patients have nonvasculitic abnormalities, with intimal hyperplasia that can progress to stenosis or aneurysmal dilatation); (3) HIV-associated vasculitis; and (4) small vessel disease [5]. Our understanding of the pathologic mechanisms of these phenotypes is incomplete. We have previously described more detailed clinicopathologic classification of HIV-associated vasculopathy [5].

Here we report the clinical, laboratory, radiologic, and autopsy features of HIV ischemic stroke patients, explore how they differ from the non-HIV ischemic stroke population, and consider the mechanisms of stroke among those starting ART.

## METHODS

### Participants

**Figure 1.**
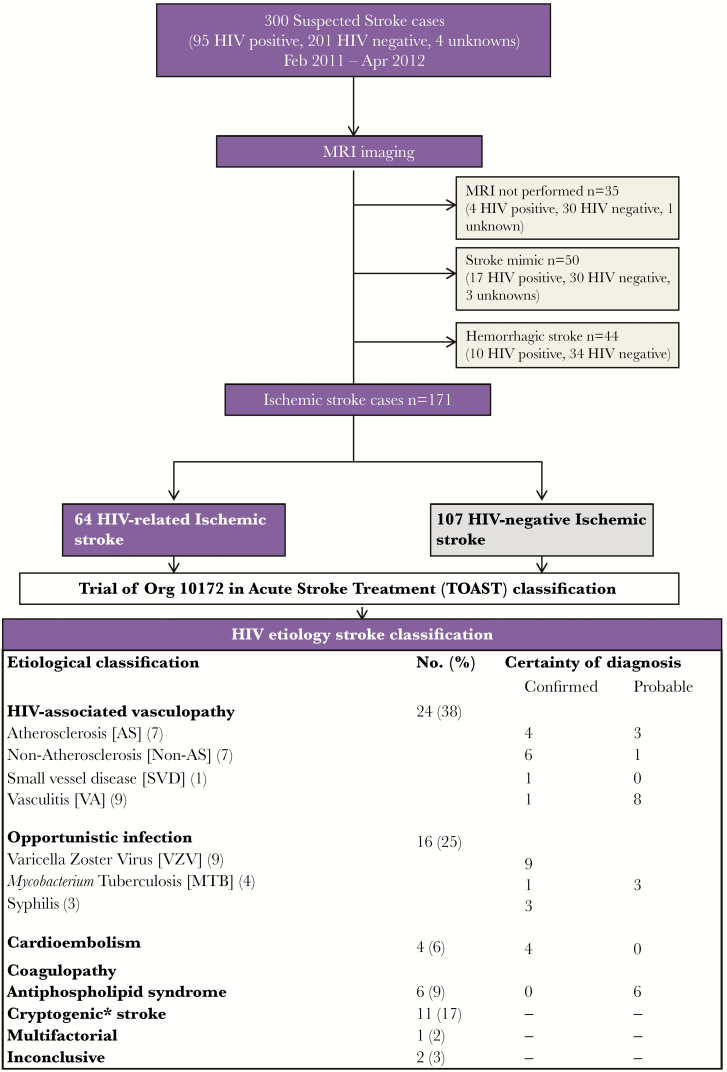
Selection procedure and classification of cases. *Noninvasive or invasive angiography has not been done and therefore the subcategory of “cryptogenic embolism” and “other cryptogenic” cannot be determined. Abbreviations: HIV, human immunodeficiency virus; MRI, magnetic resonance imaging.

The study was conducted at the Queen Elizabeth Central Hospital, Blantyre, Malawi; a large government hospital for much of Southern Malawi. The national prevalence of HIV in adults is 10.6% but higher (20%) in Blantyre [6]. Adults (≥18 years of age) who presented to the hospital within 7 days of symptom onset, and met the World Health Organization case definition of stroke—“a clinical syndrome consisting of rapidly developing clinical signs of focal (or global in case of coma) disturbance of cerebral function lasting >24 hours or leading to death with no apparent cause other than a vascular origin” [7]—were recruited to the study between February 2011 and April 2012 (Figure 1).

### Procedure and Etiological Definitions

Clinical features and potential vascular risk factors (ie, age, sex, family history, ART use, hypertension, diabetes, hypercholesterolemia, acute infection, abdominal obesity, alcohol, smoking, substance use, and previous stroke/transient ischemic attack) were recorded. Stroke severity at baseline was assessed with the National Institutes of Health Stroke Scale, and performed within 7 days of symptom onset [8, 9]. Evidence of peripheral vascular disease was determined by measuring ankle brachial index using a handheld vascular Doppler (HI Dop, Ana Wiz Ltd, United Kingdom) [10]. Magnetic resonance imaging of the brain was performed within 7 days of admission. The definitions for risk factors, severity of stroke, and imaging protocol were previously reported [2].

Investigations included full blood count, total cholesterol, random glucose, HIV-1 serology and viral load, and CD4^+^ T-lymphocyte count (CD4^+^ count) using previously described methods [2]. HIV-1 RNA load was measured using the Hologic Aptima HIV-1 Quant Dx assay (Hologic Inc, Manchester, United Kingdom). This sensitive assay has a lower limit of quantitation (LLOQ) of 30 copies/mL and a limit of detection of 13 copies/mL. Antiphospholipid syndrome diagnostics (anticardiolipin antibody, lupus anticoagulant, anti-β_2 –_glycoprotein 1; Cambridge Life Sciences, Cambridgeshire, United Kingdom), and specific infection screening—(1) serum syphilis treponemal immunoassay plus agglutination test and nontreponemal tests, and, if positive, cerebrospinal fluid (CSF) venereal disease research laboratory test, and (2) monoclonal intrathecal varicella zoster (VZV) antibody determination [11]—were not done locally and thus were performed using standard protocols, at the hematology department, Royal Liverpool Hospital, and Public Health England, respectively. All blood cultures and CSF diagnostics (ie, microscopy, biochemistry, India ink and acid-fast bacilli stains, cryptococcal antigen, standard bacterial culture, *Mycobacterium tuberculosis* [TB] culture) were performed locally at the Malawi-Liverpool-Wellcome Trust (MLW) laboratory. MLW laboratory participates in internationally recognized quality control programs including the UK National External Quality Assessment Service and the South African National Health Laboratory Service scheme. Chest radiography, electrocardiography, carotid/vertebral duplex ultrasonography, and echocardiography were also performed. When possible, a brain-only autopsy was performed in deceased HIV-infected patients. Brain tissue was stored in 10% formalin and processed at the University of Edinburgh, United Kingdom. The tissue sections were stained with hematoxylin and eosin and Ziehl-Neelsen stain. Additional staining included p24 antigen (for HIV) and CD8, CD68, and CD3 antibodies (for inflammation). The results were interpreted by a neuropathologist and general pathologist with expertise in HIV infection. Although the pathologists were not blinded to the HIV status, a consensus had to be reached among these senior pathologists, with the third (HIV pathologist) arbitrating when needed.

The etiology was determined using the Trial of Org 10172 in Acute Stroke Treatment (TOAST) classification (Figure 1). To expand the other determined and undetermined category and handle multiple etiologies in the HIV cohort, we also used the HIV Etiology for Ischemic Stroke classification, as described previously (Figure 1). The physician who determined the final diagnosis was blinded to ART status, degree of immunosuppression, and HIV viral burden.

### Ethical Consideration

The study was approved by the Liverpool School of Tropical Medicine, United Kingdom, and the College of Medicine Research Ethics Committee, University of Malawi. All participants or guardians gave written informed consent.

### Statistical Analysis

Discrete variables were reported as absolute counts and percentages. Continuous variables are shown as the median with interquartile range (IQR). HIV RNA load below the LLOQ was coded as 30 copies/mL (the assay’s quantification limit). This was then log-transformed to compare the median HIV RNA load across specified groups. Contingency tables comparing (1) HIV infected/uninfected and (2) HIV etiology for stroke classification were analyzed with Fisher 2-sided exact test. Kruskal-Wallis nonparametric analysis of variance was used to compare continuous variables.

Statistical analyses were done with Stata software version 11.2 and GraphPad Prism version 6 (GraphPad Software). A significance level of <.05 was used throughout.

## RESULTS

Three hundred patients with suspected stroke were screened during the study period, of whom 171 (64 HIV infected and 107 HIV uninfected) had ischemic strokes and met the entry criteria (Figure 1). Table 1 describes the demographic and clinical characteristics of the HIV-infected and -uninfected individuals. The 64 HIV-infected ischemic stroke cases form the basis of this study. Of these, 26 (40%) were on ART and 4 had brain autopsy.

**Table 1.** Clinical, and Radiologic Characteristics of Ischemic Stroke in Human Immunodeficiency Virus–Infected and –Uninfected Cohorts

Characteristic	HIV Infected (n = 64)	HIV Uninfected (n = 107)	*P* Value^a^
Median age, y (IQR)	40 (32–51)	66 (53–77)	<.001
Male sex	29 (45)	49 (46)	1.000
Family history	9 (14)	17 (17)	.403
Hypertension	27 (42)	89 (83)	<.001
Diabetes	2 (3)	14 (13)	.032
Hypercholesterolemia	4 (7)	10 (10)	.510
Current smoker	6 (9)	26 (24)	.016
Recent infection	12 (19)	8 (8)	.082
Alcohol intake	13 (21)	14 (13)	.379
Cannabis use	1 (2)	3 (3)	1.000
Obesity			.496
Tertile 1	14 (22)	17 (16)	
Tertile 2	23 (37)	33 (31)	
Tertile 3	26 (41)	56 (53)	
Median ankle brachial index (IQR)	1.01 (0.96–1.01)	1.01 (0.94–1.06)	.946
Previous TIA	4 (6)	7 (7)	.274
Previous stroke	4 (6)	15 (14)	.244
Radiologic characteristics			
Acute/subacute MRI lesions			
Cerebral cortex^b^	39 (68)	61 (66)	.721
Cerebellum	4 (7)	3 (3)	.297
Brainstem	6 (11)	9 (10)	.866
Basal ganglia	39 (68)	39 (42)	.002
Periventricular white matter disease	24 (42)	33 (36)	.418
Other^c^	1 (2)	2 (2)	.162
>1 focal lesion	12 (21)	6 (8)	.034
Stroke characteristics			
Median NIH stroke scale (IQR)	12 (8–14)	11 (7–18)	.813
Etiology of stroke (TOAST)			<.001
Large artery disease	14 (21)	14 (10)	
Cardiothromboembolism	4 (6)	13 (9)	
Small vessel disease	1 (1)	3 (2)	
Stroke of other determined cause^d^	31 (46)	15 (11)	
Stroke of undetermined cause	18 (27)	92 (67)	
Hospital fatality	11 (17)	10 (9)	.152

Data are presented as No. (%) unless otherwise indicated.

Abbreviations: HIV, human immunodeficiency virus; IQR, interquartile range; MRI, magnetic resonance imaging; NIH, National Institutes of Health; TIA, transient ischemic attack; TOAST, Trial of Org 10172 in Acute Stroke Treatment.

^a^Categorical variables were analyzed with Fisher 2-sided exact test. Kruskal-Wallis nonparametric analysis of variance was used to compare continuous variables.

^b^Cerebral cortex includes frontal, temporal, occipital, and parietal lobe.

^c^Corpus callosum, hypothalamus, pituitary, craniocervical junction.

^d^Stroke of other determined cause in the HIV-uninfected group includes probable antiphospholipid syndrome (7), syphilis (4), varicella zoster (2), tuberculosis (1), probable vasculitis (1).

^e^See Table 2 for HIV-associated stroke.

### Comparison Between HIV-Infected and -Uninfected Patients

Compared with the HIV-uninfected patients, the HIV-infected patients were significantly younger (median age, 40 years vs 66 years; *P* < .001) and less likely to be hypertensive (42% vs 83%; *P* < .001; Table 1). Other vascular risk factors, including diabetes, hypercholesterolemia, and being a smoker, were more common in patients who were HIV uninfected. Imaging analysis showed that basal ganglia ischemia occurred more often with HIV ischemic stroke (68% vs 42%; *P* < .001). Using the TOAST classification, the other determined and undetermined etiologic category were common in both groups. However, for the better characterized categories, large artery disease had a different distribution and occurred more frequently in the HIV-infected group (21% vs 10%; Table 1).

### Etiology of HIV-Related Ischemic Stroke

HIV-associated vasculopathy (ie, accelerated atherosclerosis, nonatherosclerotic vasculopathy, HIV-associated vasculitis, and small vessel disease) was the commonest etiology (38%), followed by opportunistic infections (25%); the latter included VZV, TB, and syphilis (Table 2). Although VZV was the most frequent infection, it was often not clinically obvious, with only 3 of 9 (30%) having had a vesicular rash in the corresponding cranial distribution within 6 months of their stroke. No patient had evidence of occult cryptococcal disease. Despite a comprehensive workup, a specific cause was not determined in the 17% with cryptogenic stroke.

**Table 2.** Clinical Features of the Different Etiologies Found in Human Immunodeficiency Virus–Related Ischemic Stroke

Feature	HIV-Associated Vasculopathy^a^ (n = 23)			Opportunistic Infections (n = 16)	Antiphospholipid Syndrome (n = 6)	Cardiothromboembolism (n = 4)	Cryptogenic^b^ Stroke (n = 11)	*P* Value^c^
	Atherosclerotic Vasculopathy (n = 7)	Nonatherosclerotic Vasculopathy (n = 7)	HIV-Associated Vasculitis (n = 9)					
Median age, y	60 (50–68)	33 (24–42)	35 (32–42)	35 (28–41)	42 (32–52)	58 (48–69)	44 (31–54)	<.001
Male sex ART status	4 (57)	4 (57)	4 (44)	5 (31)	4 (67)	2 (50)	2 (50)	.606
Untreated	4 (57)	4 (57)	2 (22)	12 (75)	4 (67)	2 (50)	7 (64)	.048
<6 mo on treatment	0	3 (43)	6 (67)	3 (19)	2 (33)	1 (25)	1 (9)	
≥6 mo on treatment	3 (43)	0	1 (11)	1 (6)	0	1 (25)	3 (27)	
CD4^+^ T-lymphocyte count, cells/μL	271 (192–318)	248 (218–305)	88 (15117)	131 (61–294)	93 (63–159)	302 (240–558)	204 (51–458)	.031
HIV blood viral load, log_10_ copies/mL	3.1 (0–4.4)	3.7 (1.5–4.3)	0 (0–2.5)	3.5 (2.4–4.6)	4.7 (2.0–5.3)	1.5 (0–4.0)	1.5 (0–4.6)	.183
Hemoglobin, g/dL	12.0 (9.0–15.0)	11.0 (9.0–15.0)	12.0 (10.0–14.0)	12.0 (10.0–13.0)	10.0 (9.0–12.0)	14.0 (13.0–15.0)	12.0 (9.0–13.0)	.720
NIH stroke scale	12 (7–14)	11 (8–17)	13 (12–18)	13 (8–16)	10 (7–12)	9 (8–11)	11 (6–14)	.596
Hospital fatality	2 (29)	1 (14)	2 (22)	3 (19)	1 (17)	0	2 (18)	.955

Data are presented as No. (%) or median and interquartile range (for continuous variables).

Abbreviations: ART, antiretroviral treatment; HIV, human immunodeficiency virus; IQR, interquartile range; NIH, National Institutes of Health.

^a^Small vessel disease (n = 1), multifactorial stroke (n = 1), and inconclusive (n = 2) were not included in the analysis.

^b^Noninvasive or invasive angiography has not been done and therefore the subcategory of “cryptogenic embolism” and “other cryptogenic” cannot be determined.

^c^Categorical variables were analyzed with Fisher 2-sided exact test. Kruskal-Wallis nonparametric analysis of variance was used to compare continuous variables.

Age (*P* < .001), CD4^+^ count (*P* = .031), and ART status (*P* = .048) differed significantly for the different etiologic groups. For example, patients with nonatherosclerotic vasculopathy, HIV-associated vasculitis, opportunistic infection, and cryptogenic stroke had a median age ≤45 years (ie, young stroke) whereas those with atherosclerotic vasculopathy and cardiothromboembolism were older (Table 2). In the most immunosuppressed patients whose median CD4^+^ count was <200 cells/μL, HIV-associated vasculitis, opportunistic infections, and antiphospholipid syndrome were the most frequently found etiologies. The ART status differed significantly for these patients: 67% of those with HIV-associated vasculitis had started ART in the 6 months prior to their stroke; in contrast, only 33% of those with antiphospholipid syndrome and 19% of those with opportunistic infections had recently started ART (Table 2). Furthermore, blood HIV viral load differed across these groups, being high (median, 3.5 and 4.7 copies/mL, respectively) in patients with opportunistic infections and antiphospholipid syndrome, in contrast to being below the lower limit of quantitation, in patients with HIV-associated vasculitis. There were no significant differences in measured CSF cell count and biochemistry across the groups.

### HIV-Associated Vasculopathy

The HIV-associated vasculopathy subtypes included accelerated atherosclerosis (n = 7), nonatherosclerosis (n = 7), and HIV-associated vasculitis (n = 9); the median age for these patients was 60 years, 33 years, and 35 years, respectively (Table 2). Because there was only 1 case of small vessel disease, this was not included in the detailed analysis. The various types of HIV-associated vasculopathy differed by ART status and CD4^+^ count (Table 2); for example, no patient with atherosclerotic vasculopathy had started ART in the last 6 months, compared with 43% of the nonatherosclerotic and 67% of the HIV-associated vasculitis subtypes.

At autopsy (Supplementary Figure 1), 2 patients showed extensive atherosclerosis in all sized vessels (the images were consistent); 1 was on ART and young (50 years), with no established vascular risk factors. Although the other patient was older (74 years), not on ART, with a new diagnosis of hypertension; the degree of atherosclerosis was marked. All patients with HIV-associated vasculitis had a median CD4^+^ count of <200 cells/μL, and HIV-1 RNA was below the LLOQ; this differed from those with nonatherosclerotic and atherosclerotic vasculopathy (Table 2).

### Initiating ART

**Figure 2.**
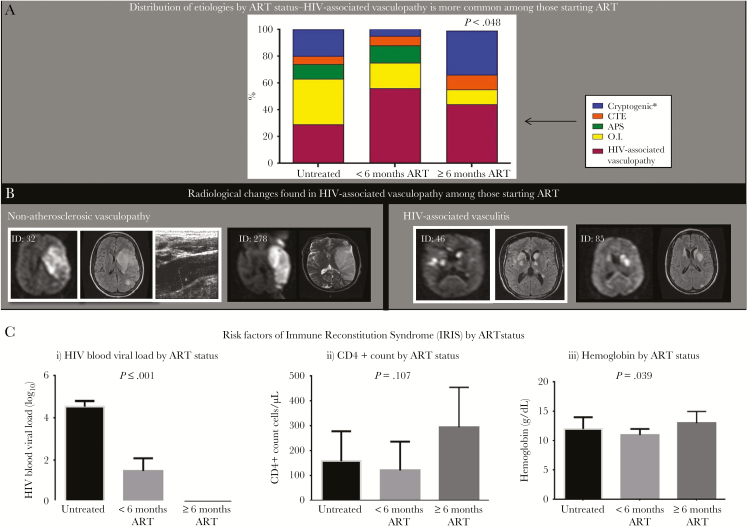
Clinical, radiologic, and laboratory features among those starting antiretroviral therapy (ART). *A*, Etiology by ART status shows human immunodeficiency virus (HIV)–associated vasculopathy to be the most common etiology among those starting ART. *B*, Radiologic examples of HIV-associated vasculopathy found among those starting ART: ID 32—diffusion-weighted (left) and fluid attenuated inversion recovery (FLAIR) (middle) sequences show a left middle cerebral artery infarct, while Doppler of the left common carotid artery (right) illustrates underlying concentric stenosis (≥70%) extending into the bulb; ID 278—middle cerebral artery infarct on diffusion-weighted (left) and T2-weighted (right) sequences; ID 46 and 85—diffusion-weighted and FLAIR sequences show multifocal ischemic lesions in the basal ganglia and cortices. *C*, Risk factors of immune reconstitution inflammatory syndrome compared across the ART groups. Kruskal-Wallis nonparametric analysis of variance was used to compare continuous variables across the ART status groups. *Noninvasive or invasive angiography has not been done and therefore the subcategory of “cryptogenic embolism” and “other cryptogenic” cannot be determined. Abbreviations: APS, antiphospholipid syndrome; ART, antiretroviral therapy; CTE, cardiothromboembolism; HIV, human immunodeficiency virus; IRIS, immune reconstitution inflammatory syndrome; O.I., opportunistic infection.

**Figure 3.**
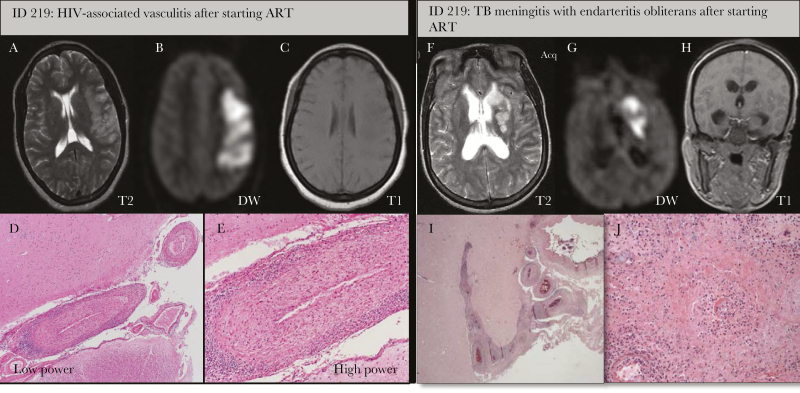
Radiohistologic characteristics in patients presenting with human immunodeficiency virus (HIV)–associated vasculitis vs vasculitis related to tuberculous meningitis after starting antiretroviral therapy (ART). *A–E*, A-32 year-old (5 months pregnant) woman on ART for <6 months with an acute right arm monoparesis, dysphasia, and headache. Her CD4^+^ count was 175 cells/μL and HIV blood and cerebrospinal fluid (CSF) viral load were undetected on admission. Mild pleocytosis (white cell count was 10 cells/μL), moderately elevated protein (1.6 mg/L), and a glucose ratio of 0.48 was found on CSF examination. *A–C*, Magnetic resonance imaging (MRI) confirmed an acute middle cerebral artery infarct. *D* and *E*, Histopathology showed multiple infarction of the cortical laminar type, marked periarteritis with foci of muscle necrosis, present in all sized arteries. There was lymphocytic meningitis but no granuloma or caseation or giant cells typical of tuberculous meningitis. There were no cytomegalovirus inclusion bodies, and varicella zoster intrathecal immunoglobulin G was negative. *F*–*J*, A 34-year-old woman on ART for <6 months with an acute right arm weakness, headache, neck ache, and fever. CD4^+^ count was 128 cells/μL and HIV blood and CSF viral load on admission were 1.48 and 3.22 log_10_ copies/mL, respectively. There was no CSF pleocytosis but a markedly elevated protein of 16.6 mg/L and CSF-to-glucose ratio of 0.28. Brain MRI confirmed an acute infarct of the basal ganglia with mild hydrocephalus. *I* and *J*, Histopathology showed endarteritis obliterans of the small arteries with and a recent infarct of the basal ganglia. There was widespread meningeal inflammation with confluent and discrete tuberculoid granulomas, typical caseating necrosis, and Langhans giant cells. There were superficial Rich foci (ie tuberculous cerebritis adjacent to the meninges). Acid-fast bacilli stain was negative but histology was characteristic of tuberculous meningitis. Abbreviations: ART, antiretroviral therapy; DW, diffusion weighted; HIV, human immunodeficiency virus; TB, tuberculosis.

Sixteen of 64 (25%) patients had an ischemic stroke within 6 months of starting ART. Ten (63%) of these recent ART initiators had a stroke within 1 month of starting ART. The median age, CD4^+^ count, and blood HIV RNA load were 37 years (IQR, 31–47), 122 (IQR, 73–237) cells/μL, and 1.5 (IQR, 0.7–2.1) log_10_ copies/mL, respectively. We explored established risk factors for immune reconstitution inflammatory syndrome (IRIS), such as anemia, low CD4^+^ count, and a drop in HIV RNA load. Patients recently started on ART had the lowest median CD4^+^ count (122 [IQR, 73–236] cells/μL compared with 159 [IQR, 65–279] cells/μL in patients never started on ART, and 295 [IQR, 192–455] cells/μL in patients on ART for ≥6 months (*P* = .107). Recent ART initiators were also more anemic (median hemoglobin, 11.0 [IQR, 9.0–12.0] g/dL) and had lower viral loads than the other ART categories (Figure 2). The distribution of etiologies differed substantially by ART status group (*P* < .048; Figure 1), in keeping with the epidemiologic evidence that the first 6 months of ART is a high risk period for stroke [2]. Within this time period, HIV-associated vasculopathy (specifically HIV-associated vasculitis [n = 6] and nonatherosclerotic vasculopathy [n = 3]) was the commonest diagnosis (56%). Brain histologic material from 2 IRIS-like cases revealed TB meningitis and HIV-associated vasculitis (Figure 3).

## DISCUSSION

This in-depth analysis of a large cohort of patients shows that ischemic stroke in people with HIV infection is distinct from the non-HIV population, with a younger age of onset and a different risk factor profile. Based on clinical, radiologic, and autopsy analysis, we found that HIV-associated vasculopathy and opportunistic infections were the most common etiologies. Among patients with HIV-associated vasculopathy, the 3 subtypes (ie, accelerated atherosclerosis, nonatherosclerosis, and vasculitis) appear to have different risk factors. Importantly, most ischemic stroke patients with nonatherosclerotic vasculopathy or HIV-associated vasculitis had recently started on ART, which is suggestive of IRIS.

IRIS occurs during immune system recovery after an immunodeficient state. It is associated with a rapid decline (of ≥2 logs) in HIV viral load, a low nadir and then rising CD4^+^ count following ART introduction, and anemia [12–15]. The mechanism, although widely believed to be driven by infiltration of active T cells, still remains unclear [14]. In our cohort, those presenting with a stroke after recently starting ART showed some risk factors for IRIS [16]. However, although HIV viral loads appeared to be largely suppressed, we did not see the higher CD4^+^ counts and therefore evidence of immune reconstitution typically associated with IRIS. This immunovirologic discordance was unexpected and suggests persistent immune dysregulation. Arguably, cell counts may not wholly reflect function and as evidenced by viral suppression, there may have been immune recovery even in the absence of an increased CD4^+^ count [15]. Such immune-virologic discordance was recently implicated in non-AIDS complications, and thus this merits further investigation [17].

Patients diagnosed with HIV-associated vasculitis were highly immunosuppressed and thus it is plausible that vessel wall inflammation was driven by an undiagnosed opportunistic infection. Tuberculosis and cryptococcus, for example, are frequent triggers of central nervous system (CNS) IRIS [18]. However, patients with confirmed opportunistic infections tended not to be on ART, and had a correspondingly high HIV viral load. Furthermore, TB was only identified in 6% of ischemic stroke and we did not detect cryptococcal disease. Of our 4 patients who had autopsy following ischemic stroke, 1 patient with characteristic features of endarteritis obliterans was diagnosed clinically and confirmed at autopsy with TB meningitis. Nonetheless, our results are more consistent with CNS-IRIS triggered by an immune response to HIV viral antigens per se [14]. The postulated mechanisms include immune response directed at residual HIV virus in the CNS, persistent release of HIV-Tat protein from HIV-infected cells despite control of viral replication, and inflammatory responses directed against self-antigens [14].

Patients with atherosclerotic vasculopathy were not as immunosuppressed as patients with other subtypes of HIV-associated vasculopathy. The relatively young patient (ID: 218), who was on ART for >6 months, showed histologic evidence of extensive atherosclerosis in the absence of other vascular risk factors (Supplementary Figure 1). At a population level, studies in high-income countries have consistently shown that HIV-infected individuals have a substantially high risk of stroke, roughly equivalent to that of general population cohorts 10–20 years older than themselves [19]. This is despite exposure to opportunistic infections being far lower, and the additive risk of ART toxicity and HIV/ART-induced metabolic dysregulation (eg, hyperlipidemia) being accounted for [4, 19]. HIV could have a causal role in this disease mechanism, but this is still open to debate. However, there is growing evidence that HIV-related chronic inflammation even in well-suppressed HIV-infected individuals is linked to subclinical vasculopathy [4]. While HIV-associated vasculopathy appears to be more common in sub-Saharan Africa compared with elsewhere, the atherosclerotic and cryptogenic subtypes are likely to become the predominant subtypes as the HIV population ages and the disease stabilizes, and thus, warrants further investigation.

There were some limitations to the study; for example, we did not screen for sickle cell disease. However, although commonly associated with stroke elsewhere in sub-Saharan Africa, the prevalence of sickle cell disease in Malawi is low (<2%) and thus unlikely to have made a major contribution [20]. The absence of cerebral angiography limited our ability to refine the diagnosis of the cryptogenic group and thus further subdivide them into cardiac embolic or noncardiac embolic causes. Indeed, the latter could have represented undiagnosed HIV-associated vasculopathy. Furthermore, nonatherosclerotic vasculopathy and HIV-associated vasculitis could be manifestations of the same disease process at different stages of HIV infection, It is possible that the hospital recruitment may have been biased against milder cases in the community. It is also possible that the risk of stroke seen in those starting ART may be related to being sick and not ART itself, although difficult to tease out; our proposed mechanism of IRIS is not dissimilar to other infections, in the very immunosuppressed, such as TB and cryptococcal CNS infection [15, 21]. Finally, CNS-IRIS is associated with a poor prognosis and is often fatal within days to weeks if untreated, leaving the possibility that ischemic stroke among those starting ART could have been underestimated if patients died before hospital admission [14, 18].

HIV-associated vasculopathy and opportunistic infections are common causes of HIV-related ischemic stroke. Furthermore, subtypes of HIV-associated vasculopathy may manifest as a result of an immune reconstitution-like syndrome after starting ART. This study highlights the different phenotypes of HIV-associated vasculopathy and ties in with emerging data on neuroinflammation before and after HIV infection. Our understanding of the underlying mechanism and the role that HIV plays is incomplete, especially on better treated cohorts with cryptogenic stroke, and possibly “HIV-associated” atherosclerosis. This highlights the importance for future mechanistic studies to underpin the pathogenesis of these various subtypes and, in time, pave the way for appropriate interventions.

## Supplementary Data

Supplementary materials are available at *The Journal of Infectious Diseases* online. Consisting of data provided by the authors to benefit the reader, the posted materials are not copyedited and are the sole responsibility of the authors, so questions or comments should be addressed to the corresponding author.

## Supplementary Material

Supplement_Fig1

Supplementary_Figure_Legend

## Notes

***Acknowledgments.*** We thank Malcolm Molyneux for his helpful input in developing the autopsy protocol; Kevin Brown, David Brown, and Catherine Ison from Public Health England for their help with VZV and syphilis diagnostics; and Colin Downey for his help with antiphospholipid syndrome diagnostics.

***Author contributions.*** L. A. B., T. S., T. J. A., R. S. H., E. L. C., and M. D. C. designed the study. L. A. B. performed the research, did the literature search, analyzed the data, and wrote the first draft of the paper. T. S., M. D. C., R. S. H., E. L. C., E. J., and S. L. revised the first draft of the paper. I. T., E. J., K. C., H. M., S. K., C. S., S. L., S. K., and M. H. performed the research. T. S. and T. J. A. supervised the conduct of the study. All authors contributed to critical revision of the manuscript.

***Financial support.*** This work was supported by a Clinical PhD Fellowship from The Wellcome Trust, Great Britain (to L. A. B.) and a United Kingdom Medical Research Council Fellowship (to T. S.), and The National Institute for Health Research Health Protection Research Unit in Emerging and Zoonotic Infections (to T. S.). The Malawi-Liverpool-Wellcome Trust Clinical Research Programme is supported by a Strategic Award from The Wellcome Trust.

***Potential conflicts of interest.*** M. C. has received lecture/meeting fees from AbbVie. All other authors report no potential conflicts. All authors have submitted the ICMJE Form for Disclosure of Potential Conflicts of Interest. Conflicts that the editors consider relevant to the content of the manuscript have been disclosed.
