# Supplementary figures and images for: The Role of Human Immunodeficiency Virus–Associated Vasculopathy in the Etiology of Stroke
Source: J Infect Dis. 2017 Jul 22;216(5):545–53. doi: 10.1093/infdis/jix340 (PMC5853476; doi:10.1093/infdis/jix340)

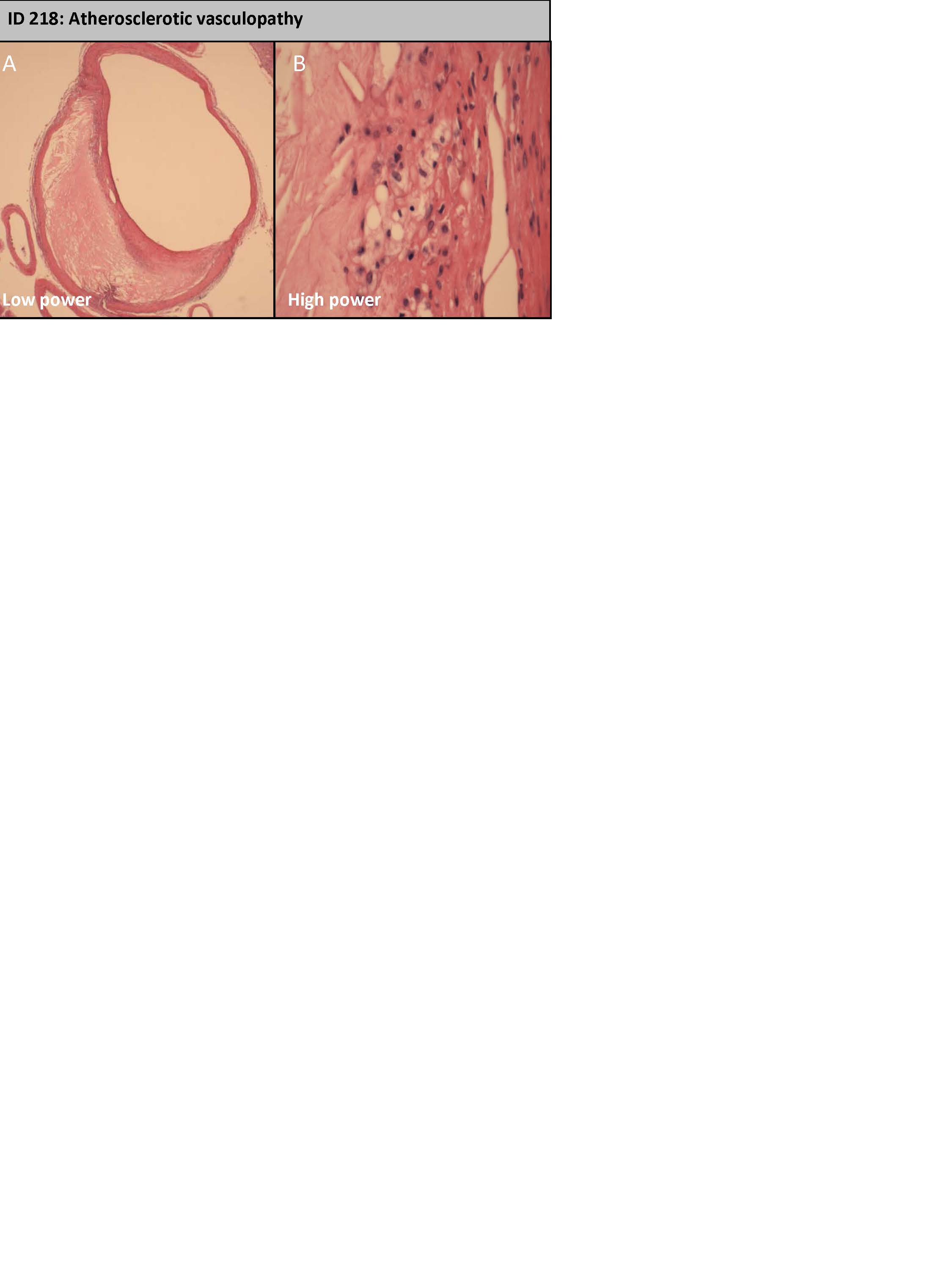

Supplement: Supplement_Fig1 [file jix340_suppl_suppl_fig1.jpeg]
