# Supplementary material for: The Role of Human Immunodeficiency Virus–Associated Vasculopathy in the Etiology of Stroke
Source: J Infect Dis. 2017 Jul 22;216(5):545–53. doi: 10.1093/infdis/jix340 (PMC5853476; doi:10.1093/infdis/jix340)
Supplement: Supplementary_Figure_Legend [file jix340_suppl_supplementary_figure_legend.docx]

**Supplementary Figure Legend**

**Supplement Figure 1: Histologic illustration of atherosclerotic vasculopathy:**

A 50 year old man on ART for greater than 6 months with an acute left hemiparesis. His CD4+ count was 192 cells/mm^3^ and HIV blood and CSF viral load were undetected on admission. He had no pleocytosis, a mildly elevated protein (0.9 mg/L) and a glucose ratio of 0.48 on CSF examination. A comprehensive etiological screen was unremarkable. Histopathology showed an acute infarct in the right cerebral hemisphere, and extensive atherosclerosis in all sized vessels, in both the left and right cerebral hemispheres. Sections of the right carotid artery had substantial atheroma. There was no acute thrombus. There was no HIV-associated encephalitis.
